# Deep Convolutional Extreme Learning Machine and Its Application in Handwritten Digit Classification

**DOI:** 10.1155/2016/3049632

**Published:** 2016-08-17

**Authors:** Shan Pang, Xinyi Yang

**Affiliations:** ^1^College of Information and Electrical Engineering, Ludong University, Yantai 264025, China; ^2^Department of Aircraft Engineering, Naval Aeronautical and Astronautical University, Yantai 264001, China

## Abstract

In recent years, some deep learning methods have been developed and applied to image classification applications, such as convolutional neuron network (CNN) and deep belief network (DBN). However they are suffering from some problems like local minima, slow convergence rate, and intensive human intervention. In this paper, we propose a rapid learning method, namely, deep convolutional extreme learning machine (DC-ELM), which combines the power of CNN and fast training of ELM. It uses multiple alternate convolution layers and pooling layers to effectively abstract high level features from input images. Then the abstracted features are fed to an ELM classifier, which leads to better generalization performance with faster learning speed. DC-ELM also introduces stochastic pooling in the last hidden layer to reduce dimensionality of features greatly, thus saving much training time and computation resources. We systematically evaluated the performance of DC-ELM on two handwritten digit data sets: MNIST and USPS. Experimental results show that our method achieved better testing accuracy with significantly shorter training time in comparison with deep learning methods and other ELM methods.

## 1. Introduction

Extreme learning machine is a novel learning algorithm for general single-hidden-layer neural networks proposed by Huang et al. [1]. In ELM, the input weights and hidden biases are randomly generated, and the output weights are analytically determined by regularized least square method, providing a simple deterministic solution. There are no iterations and parameters tuning as in back propagation (BP) based neural networks (NNs). Furthermore, solving the regularized least squares in ELM is also faster than solving the quadratic programming problem in standard support vector machine (SVM) method. Studies have proved that ELM learns much faster with higher generalization performance than NNs or SVM [2].

Due to its extreme fast training and good generalization performance, ELM has been becoming a significant research topic for pattern recognition and machine learning. ELM and its variant methods present competitive accuracy with superb efficiency in many pattern recognition applications such as face recognition [3, 4], engine fault diagnosis [5], hyperspectral images classification [6], and human action recognition [7, 8]. However, due to their shallow architectures, feature learning using ELM methods may not be effective for some image classification applications, even with a large number of hidden nodes.

In recent years, some deep learning methods have been highlighted and show promising results and significantly outperform shallow neural networks in the field of image classification [9–12]. Composed of many layers, deep learning methods gradually extract more complicated and invariant features from the raw input images than shallow neural networks [13]. The emergence of many large-scale data sets and more powerful computing environments has made the training of deep neural networks possible, leading to a widespread application of deep learning methods.

Among these methods, convolutional neural network (CNN) has gained incredible popularity in many different domains. It is even becoming the default option for difficult tasks on large image data sets. With local receptive field (LRF) and shared weights, CNN is able to take advantage of the 2D structure of input images and has fewer parameters than fully connected deep networks with the same number of hidden nodes; thus it is easier to train. As all the hidden nodes in CNN need to be tuned with BP learning method, CNN learning faces the problems inherited from BP algorithm such as local minima and time-consuming and intensive human intervention.

On the contrary, ELM does not need tuning of parameters and is extremely fast to implement. Therefore Huang combines the concept of LRF with ELM and proposed a local receptive field based extreme learning machine (LRF-ELM) [14] in order to learn local correlations of input images. The input layer and hidden convolution layer in LRF-ELM are locally connected which allows the network to consider local structures of images. Results on NORB data set show that it has better performance than standard CNN and DBN.

Since LRF-ELM has only one convolution layer followed by a pooling layer, the performance is restricted by its shallow architecture. Another problem is that many feature maps are required in its convolution and pooling layer to attain good performance. Therefore, LRF-ELM consumes much computer memory in implementation. To solve these problems, in this paper, we propose a deep convolutional extreme learning machine (DC-ELM). It adopts multiple alternate convolution layers and pooling layers to obtain more abstract and meaningful feature representations than LRF-ELM. Different from CNN, the local receptive weights are randomly generated without tuning and the output weights are analytically calculated. In order to save computer memory and training complexity, it adopts stochastic pooling [15] in the last hidden layer to reduce dimensionality of feature vector.

To verify the effectiveness of the proposed algorithm, we applied it to some handwritten digits classification tasks and compared it with other state-of-the-art methods. Handwritten digits' recognition has its real world application, such as the postal mail sorting or form data processing [16]. Several methods based on neural networks [17–19], machine learning [20, 21], and other techniques [22, 23] have been studied. Recently some ELM based methods have also been applied to handwritten digits recognition and show good performance on MNIST data set. The ML ELM proposed in [24] achieved 99.03% correct classification. And a test accuracy of 99.19% by deep ELM is achieved in [25]. In [26] a RF-C-ELM was proposed and attained a test accuracy of 99.43%, very close to 99.61%, obtained by Deep Conv. Net [27].

The rest of the paper is organized as follows.  Section 2 gives a brief review of ELM and LRF-ELM.  Section 3 describes the proposed DC-ELM. In Sections 4 and 5, our method is applied to MNIST and USPS data sets and compared with other state-of-the-art methods.  Section 6 analyzes the effect of stochastic pooling and some other parameters. Finally, Section 7 draws the conclusions and points out the future work.

## 2. Reviews of ELM and LRF-ELM

### 2.1. Extreme Learning Machine (ELM)

ELM was proposed for single-hidden layer feedforward neural networks (SLFNs). It is very different from conventional neural network learning algorithms. It randomly chooses the parameters of hidden nodes and analytically determines the output weights. Thus the training is extremely fast and efficiently completed without time-consuming iterations.

The input data is mapped to an *L*-dimensional ELM random feature space, and the network output is

(1) $$\begin{array}{c} f_{L}\left(\;x\;\right)=\overset{L}{\underset{i=1}{\sum }}\beta_{i}g_{i}\left(\;x\;\right)=h\left(\;x\;\right)\beta , \end{array}$$

where **β** = [**β**_1_, **β**_2_,…, **β**_*L*_]^*T*^ is the matrix of output weights and **h**(**x**) = [*g*_1_(**x**),…, *g*_*L*_(**x**)]^*T*^ are the hidden node outputs for input **x**. *g*_*i*_(**x**) is the output of *i*th hidden node. Given *N* training samples (**x**_*i*_, **t**_*i*_), the ELM can approximate these *N* samples with zero error which means that

(2) $$\begin{array}{c} H\beta =T \end{array}$$

**H** = [**h** ^*T*^(**x**_1_),…, **h** ^*T*^(**x**_*N*_)]^*T*^, and **T** = [**t**_1_, **t**_2_,…, **t**_*N*_]^*T*^ are the matrix of desired output. The output weights can then be calculated using regularized least squares method as follows:

(3) $$\begin{array}{c} \beta ={\left(\;\frac{I}{C}+H^{T}H\;\right)}^{-1}H^{T}T, \end{array}$$

where *C* is the regularization parameter, which is used to obtain better generalization performance.

### 2.2. Local Receptive Fields Based Extreme Learning Machine (LRF-ELM)

As the name suggests, LRF-ELM introduces local receptive field to the input layer, thus obtaining a locally connected ELM. The hidden layers of LRF-ELM consists of a convolution layer and a pooling layer. They are composed of several feature maps. The input weights between input and convolution layers are first randomly generated according to some continuous probability distribution and then orthogonalized in order to obtain a more complete set of features. The square root pooling is used to formulate the combinatorial node in pooling layer. The square and summation operations introduce rectification nonlinearity and translational invariance, respectively, into the network, which is very important for successful image processing tasks. The pooling layer is in full connection with the output layer. The output weights are analytically calculated as in the unified ELM using regularized least squares.

LRF-ELM with local randomly connected hidden nodes can be regarded as a specific type of ELM. Huang has proved that the universal approximation/classification capability of such LRF-ELM can still be preserved.

## 3. Deep Convolutional Extreme Learning Machine

In this section, we propose a new deep convolutional extreme learning machine designed to solve image classification tasks. DC-ELM combines the feature abstracting performance of convolutional neuron network and fast training of extreme learning machine.

**Figure 1:**
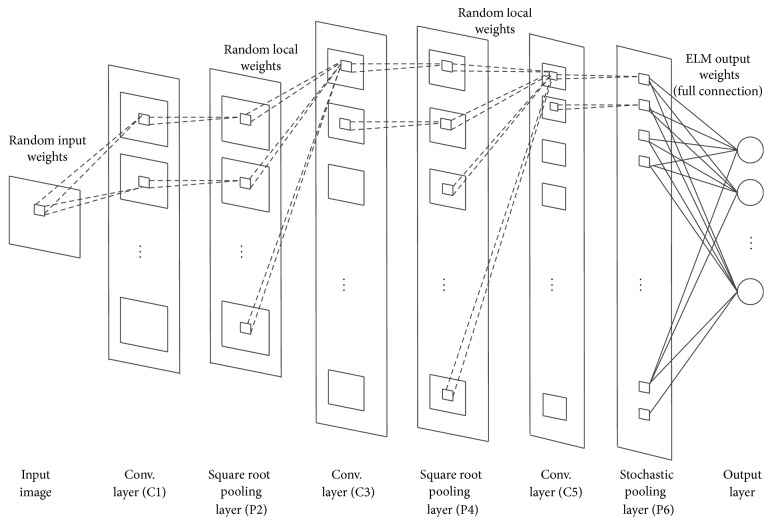
An example of DC-ELM network (with three convolution layers).

As shown in Figure 1, the structure of DC-ELM consists of an input layer, an output layer, and several hidden layers which are arranged alternately as one convolution layer followed by one pooling layer. The convolution layer consists of several feature maps which are grouped by convolution nodes. The input weights of the same feature map are shared while being distinct among different maps. The square root pooling layer is used to introduce translational invariance to the network. It has the same number of feature maps with the same size as the previous convolution layer. The node in any feature map of a convolution layer is connected to all the feature maps in its previous pooling layer, while the node on a feature map in pooling layer is connected to only one corresponding feature map in its previous convolution layer as shown in Figure 1. The last pooling layer adopts stochastic pooling strategy, thus reducing the size of its feature maps. It is in full connection with the output layer.

This network is designed under three considerations. First, the multiple hidden convolution and pooling layers can extract high level features effectively from input images which is key to image classification tasks. Secondly the shared local receptive weights enable our method to learn local correlations in images and handle image rotation invariance properly. Thirdly the batch training of ELM makes our method run much faster than deep learning methods.

The training procedure for DC-ELM can be described in two phases: (1) convolution feature abstracting, where DC-ELM generates high level feature layer by layer; (2) ELM classifier calculation, where all features generated by convolution and pooling layers are combined into a vector. And the output weights are analytically calculated using regularized least squares method.

### 3.1. Feature Abstraction

#### 3.1.1. Generation and Orthogonalization of Local Weights

DC-ELM randomly generates input weights between input layer and the first convolution layer (also the local weights between the pooling layer and the following convolution layer) according to some continuous probability distribution. In our paper, we choose Gaussian probability function as the sampling distribution for input weights.

The input/local weights are calculated as follows.

Generate the initial weight matrix ${\hat{A}}_{ini}$ using Gaussian probability distribution for each feature map in the convolution layer:

(4) A^ini∈Rr2×K=a^ini1,a^ini2,…,a^iniKa^inik∈Rr2,  k=1,…,K,

where *r* × *r* is the size of local receptive field; if the previous layer size is *d* × *d*, the size of the feature map would be (*d* − *r* + 1)×(*d* − *r* + 1). Then the initial weight matrix is orthogonalized using singular value decomposition method. Each column ${\hat{a}}^{k}$ of the orthogonalized weight matrix $\hat{A}$ is then transformed to **a** ^*k*^ ∈ *R* ^*r*×*r*^, the input weight to the *k*th feature map.

#### 3.1.2. Convolution

The convolution layer extracts features using the convolution operation on the input image or the feature maps in the previous pooling layer. For the convolutional node at coordinate (*i*, *j*) on the *k*th feature map in the first convolution layer, it is calculated as

(5) ci,j,kx=∑m=1r ∑n=1rxi+m−1,j+n−1·am,nki,j=1,…,d−r+1,

where **x** is the input image. Unlike CNN, the feature maps are not applied to nonlinear function. While for the map in higher level convolution layer, as it is connected to all the feature maps in previous pooling layer, we first calculate the convolution with each feature map using its local weights as (5); then we add them up, and we obtain the pooled feature map.

#### 3.1.3. Pooling

In DC-ELM, we adopt two pooling strategies: square root pooling and stochastic pooling. The effectiveness of square root pooling has been testified by some research [28, 29]. In [28], it has been shown that convolutional square pooling architectures can be inherently frequency selective and translation invariant, even when initialized with random weights. Square root pooling includes the square operation and summation operation. These two operations, respectively, introduce rectification nonlinearity and translational invariance into the network, which have been discussed in [29]. And experimental results show that square root pooling helps the network outperform max or average pooling.

The square root pooling is applied to all pooling layers except the last pooling layer. The node at coordinate (*p*, *q*) on the *k*th pooling map is calculated as

(6) hp,q,k=∑i=p−ep+e∑j=q−eq+eci,j,k2p,q=1,…,d−r+1;  if  i,j  is  out  of  bound:  ci,j,k=0,

where *e* is the pooling size.

As the size of square root pooling map maintains the same as the previous convolution layer, to reduce the dimensionality of the last hidden layer, there are two conventional choices: mean pooling and max pooling. The former takes the arithmetic mean of the nodes in each pooling region while the max pooling selects the largest node. But these two types of pooling have their own drawbacks when training deep convolutional networks. Average pooling has the effect of downweighting strong activations and leads to small pooled responses in some cases while max pooling tends to overfit the training set and affect the generalization performance. To overcome these drawbacks, a stochastic pooling scheme was proposed in [15]. It has the advantages of max pooling, but its stochastic nature helps to prevent overfitting problem. Experimental results show that stochastic pooling has better performance on image classification applications than mean and max pooling. In this work, we use the stochastic pooling scheme for the last pooling layer.

In stochastic pooling, the pooled maps are determined by sampling from each pooling region using a multinomial distribution. We first calculate the probabilities for each pooling region *R*_*j*_ by normalizing the values of the nodes within the region in previous convolution layer as follows:

(7) $$\begin{array}{c} p_{i}=\frac{c_{i}}{\sum_{n\in R_{j}}c_{n}}. \end{array}$$

Then we sample from the multinomial distribution based on *p* to pick a location *l* within the region. The pooled value *s*_*j*_ is then simply the value of node at location *l*  (*c*_*l*_):

(8) $$\begin{array}{c} s_{j}=c_{l}\;\text{where }l\sim P\left(p_{1},\ldots ,p_{\left|R_{j}\right|}\right) \end{array}$$

**Figure 2:**
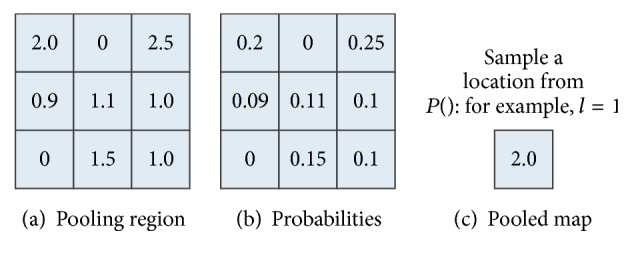
Illustration of stochastic pooling.

The procedure of stochastic pooling can be illustrated by Figure 2.  Figure 2(a) shows a pooling region with 3 × 3 elements.  Figure 2(b) is the calculated probabilities for this region. We sample from the multinomial distribution to pick a location *l* from [1,2,…, 9]; then the pooled value is the element in this location. For example, if *l* = 1, the pooled value would be 2.0, the value in the first grid of the pooled region.

It should be noted that the selected node for the pooling region may not be the largest one. Stochastic pooling can thus represent multimodal distributions of convolution nodes within a region. Furthermore, by stochastic pooling, we can obtain a pooled feature map with much smaller size than square root pooling

All the alternate convolution and pooling layers form a hierarchical feature extractor that maps the original input images into high level features which makes the classification more efficient. And the use of stochastic pooling helps DC-ELM to reduce computation burden greatly, thus leading to shorter training time.

### 3.2. ELM Output Weights Calculation

After feature generation, the values of all nodes in the last stochastic pooling layer are concatenated into a row vector. And putting the rows of *N* input samples together, we obtain the hidden layers output matrix **H** ∈ *R* ^*N*·*K*·*r*_*s*_^2^^, where *r*_*s*_ is the size of feature map in stochastic pooling layer.

Then the output weights are analytically determined using regularized least squares method as

(9) $$\begin{array}{c} \beta =\left\{\begin{array}{cc} H^{T}{\left(\frac{I}{C}+HH^{T}\right)}^{-1}T & \text{if }N\leq K\cdot r_{s}^{2},\\ {\left(\frac{I}{C}+H^{T}H\right)}^{-1}H^{T}T & \text{if }N>K\cdot r_{s}^{2}, \end{array}\right. \end{array}$$

where *T* is the labels of the input sample images and regularization parameter *C* is used to control the trade-off between the norm of output weights and training error term. The suitable setting of this parameter helps to improve the algorithm's generalization performance.

## 4. Evaluation on MNIST Data Set

In this section, we evaluate the performance of DC-ELM on MNIST [30] data set. The performance of DC-ELM is compared with ELM, LRF-ELM, and two deep learning algorithms: CNN and DBN. The MNIST handwriting data set contains 60 000 training images and 10 000 testing images of handwriting digits 0–9. As different digits have their unique shapes and different people write the number in their own ways, the MNIST is an ideal data set and commonly used to test deep learning algorithms.

### 4.1. Parameter Setting

For basic ELM, we adopt a single hidden layer with 3000 hidden nodes and sigmoid function. The parameter setting for LRF-ELM, DC-ELM, and CNN is listed in Table 1. For LRF-ELM, we employ 30 feature maps. The kernel size is 3 × 3 and the pooling size is 5 × 5. While for DC-ELM, we adopt the architecture of three convolution layers and three pooling layers. The number of feature maps in convolution layers is 5, 10, and 15 separately. And the kernel size is 3 × 3 for all convolution layers. The pooling size is 5 × 5 for the first two pooling layers and the last one adopts stochastic pooling with a pooling size of 2 × 2. The regularization parameter *C* is chosen as 0.01 by experiments for all ELM methods.

To compare fairly, CNN employs the same number of hidden layers and feature maps as DC-ELM. But unlike DC-ELM, whose feature maps size does not decrease after square root pooling, CNN adopts mean pooling and the size of feature maps will decrease after pooling. Thus the kernel or pooling size setting in CNN is different from that in DC-ELM. The kernel size is 5 × 5 for the first two convolution layers and 3 × 3 for the last one. The pooling size is 2 × 2 for all mean pooling layers. The learning epochs are set as 100. For DBN, the hidden layer structure is 500-500-1000; the learning rate is set as 0.1. The unsupervised pretraining epochs are set as 50 and supervised fine-tuning epochs are set as 100. The training data set is divided into minibatches, each containing 100 samples.

All simulations have been made in MATLAB R2008a environment running on a PC with 3.4 GHz CPU with 2 cores and 4 GB RAM.

### 4.2. Experimental Results

As our computer has only 4 GB RAM, more training samples may cause “run out of memory” error; we did not use all 60000 training samples. Instead, the training samples are randomly selected from original training set. We trained all methods with 10000 and 15000 training samples separately.

All the methods are run 10 times separately for each case. Tables 2 and 3 list the results of these methods on two cases. These results include training time, mean, and standard deviation of training/testing accuracy.

It can be seen from the tables that our method achieved the highest mean testing accuracy among all the methods on both cases with different training samples. Because we did not use all the 60000 samples for training, the testing accuracy is less than that in some literature introduced in Section 1, but it is still satisfied considering that the number of training samples is relatively small. In general, LRF-ELM achieved better testing performance than deep learning methods. Although ELM attained the highest training accuracy, its testing accuracy is not satisfying compared to other methods. It can be also found that our method consumed fewer training time on two cases than ELM or LRF-ELM. This is mainly attributed to the application of stochastic pooling which reduces the dimensionality greatly of the feature vector. On the contrary, ELM and LRF-ELM need a large amount of hidden nodes to attain good generalization performance, thus consuming more computational resource and training time. The training times of CNN and DBN are much longer than ELM methods due to the iteration nature of deep learning.

**Figure 3:**
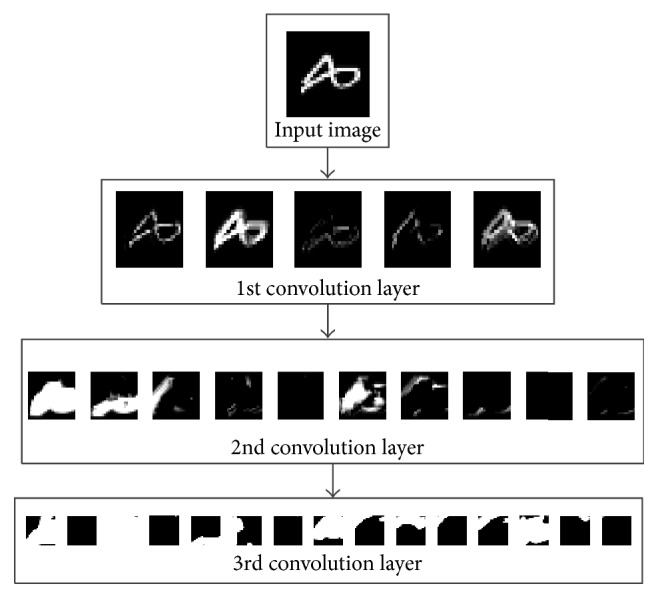
Input image and corresponding feature maps in different convolution layers.

To give insight into how DC-ELM abstract features layer by layer, Figure 3 uses one image sample (digit 8) for illustration. It displays all the feature maps generated in each convolution layer. It can be observed that the five feature maps in the first convolution layer looks similar, as they all are generated from the same input digit. However, each feature map has its unique highlighted part; thus we obtain the diverse representations of the original image. Then these feature maps are processed by pooling and passed to the next level convolution layer. After several convolution and pooling operations we can obtain the high level features which make the subsequent ELM classification more accurate and efficient.

## 5. Evaluation on USPS Data Set

We also demonstrate the performance of DC-ELM on USPS [31] data set. The USPS handwriting data set consists of 11 000 samples of handwriting digits 0–9 which are collected from different writers.

### 5.1. Parameter Setting

The parameter setting in this section is different from that on MNIST data set. We have found that DC-ELM attains better performance with fewer hidden layers for USPS than for MNIST, partly because the size of sample image in USPS data set is much smaller.

The parameter setting for LRF-ELM, DC-ELM, and CNN is listed in Table 4. Basic ELM and LRF-ELM have the same parameter setting as in Section 4 while DC-ELM adopts two hidden convolution layers with 10 and 15 feature maps separately. And the kernel size is 3 × 3 for both convolution layers. The pooling size is 5 × 5 for the first pooling layer and 2 × 2 for the second one. The regularization parameter *C* is 0.01 for all ELM methods. To compare fairly, CNN also adopts two convolution layers. The kernel size is 3 × 3 for the first convolution layer and 2 × 2 for the second. The pooling size is 2 × 2 for both mean pooling layers. The learning epochs is set as 100. For DBN, the hidden layer structure is 500-1000 and other parameters are the same as that for MNIST data set.

### 5.2. Experimental Results

We test all the methods on two different cases with 7000 and 10000 training samples separately. The training samples are randomly selected from USPS data set. We trained all methods with 10000 and 15000 training samples separately. All the methods are run 10 times separately for each case. The results are listed in Tables 5 and 6.

It can be seen from Tables 5 and 6, like on MNIST data set, that our method achieved the best testing accuracy and the shortest training time on both cases on USPS data set. ELM achieved very high training accuracy, but its performance on test data is inferior to DC-ELM. This suggests that ELM is suffering from overfitting in the training set. Also it can be concluded that deep architecture would be helpful to improve ELM's generalization ability. We can also see that training our method is much faster than LRF-ELM. And the training time of LRF-ELM is shorter than ELM. It can be concluded that the introduction of local receptive field and stochastic pooling would help to accelerate the training of ELM. The testing performance of deep learning methods is better than ELM but is inferior to LRF-ELM and DC-ELM and is much time-consuming.

**Figure 4:**
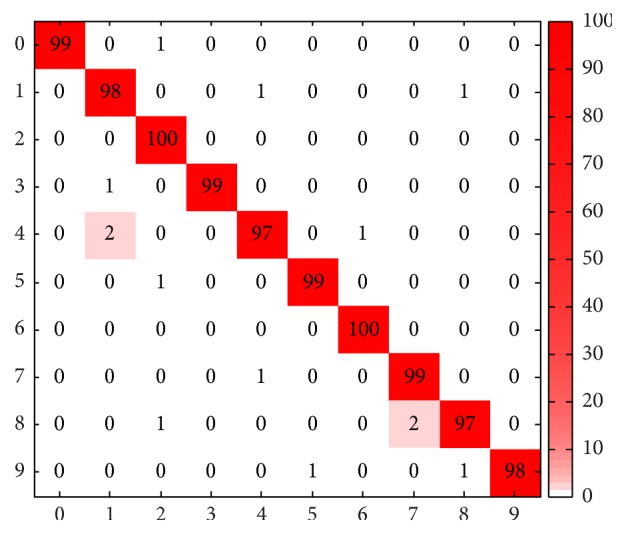
Confusion matrix obtained by DC-ELM on USPS data set.

Figure 4 shows a confusion matrix obtained by our method in a typical run; it can be seen that DC-ELM obtained a satisfying classification result since the confusion matrix is very close to dominantly diagonal. And two digits are classified with 100 percent accuracy.

## 6. Parameter Analysis

### 6.1. Effect of Network Depth

**Figure 5:**
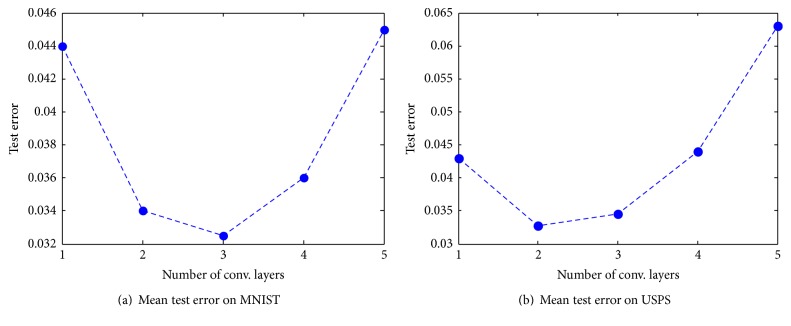
Effect of network depth on the mean test error.

The network structure has great impact on method. To study how the depth of the network affects the performance of DC-ELM, we conducted experiments on MNIST (with 15k training samples) and USPS (with 10k training samples) data sets with varying number of convolution layers, and each layer contains 10 feature maps.  Figure 5 depicts how the mean test error of ten trials varies with number of convolution layers.

Interestingly, we found that it is not “the more the better” for the hidden convolution layers. And the best depth of network is not the same for different data sets. As it can be seen in Figure 5, DC-ELM obtains best test error with three convolution layers on MNIST data set, while it performs best with only two convolution layers on USPS data set. It can be concluded that the best network structure for DC-ELM is related to applications. There is no unified optimal network structure for all data sets.

### 6.2. Effect of Regularization Parameter *C*

**Figure 6:**
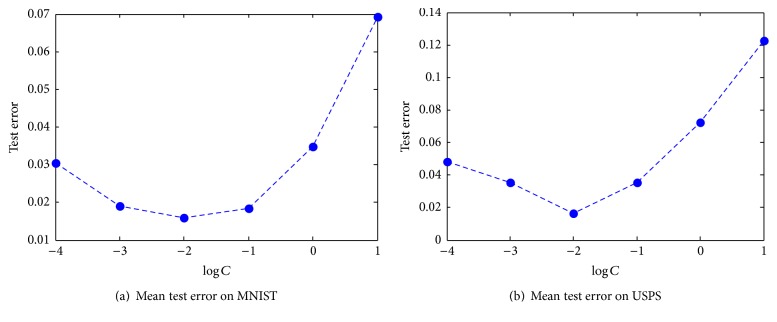
Effect of regularization parameter *C* on the mean test error.

We have also tested the effect of regularization parameter *C* in (9). It is used to control the trade-off between the norm of output weights and training error term.  Figure 6 shows how the mean test error on two data sets varies with parameter *C*. It can be seen from the figure that the test error decreases firstly and then increases greatly with parameter *C*. It reaches the lowest value when log⁡*C* = −2. Thus, in Sections 4 and 5, we set this parameter equal to 0.01.

### 6.3. Effect of Stochastic Pooling

**Figure 7:**
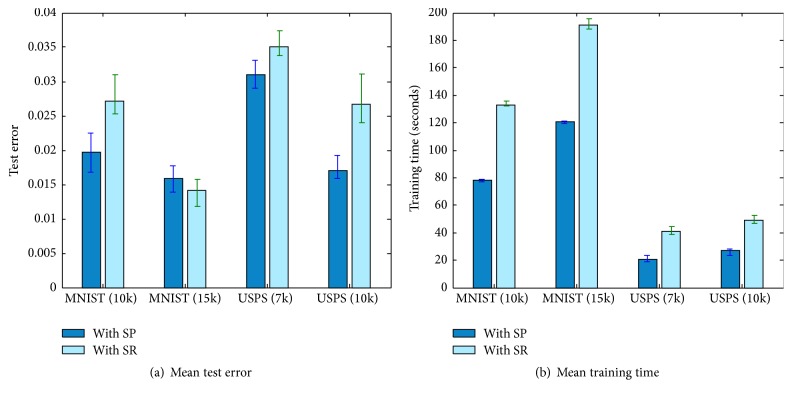
Comparison of stochastic pooling with square root pooling.

We have replaced stochastic pooling in the last pooling layer with square root pooling and tested it on MNIST and USPS.  Figure 7 shows the mean training time and test error of ten trials by square root pooling and that by stochastic pooling on MNIST and USPS data sets. The tests were conducted with different number of training samples.

It can be seen that the mean test errors with stochastic pooling are smaller or very close to that with square root pooling. This suggests that stochastic pooling is effective in improving ELM on its testing accuracy. Meanwhile the training time of DC-ELM reduces greatly in all cases. This is because of the fact that the stochastic pooling reduces the dimensionality in the last pooling layer, thus speeding up the calculation of output weights.

## 7. Conclusions

In this paper, a deep convolutional extreme learning machine is presented. It employs multiple alternate convolution layers and pooling layers that gradually extract more sophisticated and robust features from the raw input images than LRF-ELM. Based on these high level features, ELM classifier on top of the hidden layers provides a deterministic solution of the output weights. There is no parameter tuning or iterations in the training process of DC-ELM; thus the training of our method is very faster than deep learning methods.

In the implementation, we use Gaussian probability function to sample the local connections. The square root pooling is used after convolution to further introduce translational invariance into the network. We apply a simple and effective stochastic pooling strategy in the last pooling layer in order to reduce the size of feature vector, thus saving much computational resource.

Experiments conducted on handwritten digit recognition tasks show that the proposed DC-ELM presents better test accuracy on different cases than ELM, LRF-ELM, and state-of-the-art deep leaning methods. This suggests that the deep convolutional feature abstraction is more efficient than the shallow one for ELM classifier. Furthermore, training our method is much faster than other compared methods thanks to stochastic pooling and the appropriate LRF size. Therefore, we argue that our method provides an effective and useful tool which may benefit handwritten recognition and other image classification tasks.

The parameter analysis also indicates that the best network structure of DC-ELM is application-dependent. Thus it may be worth investigating method which determines the optimal network structure for different image classification applications in the future.

## Figures and Tables

**Table 1:** Parameter setting for LRF-ELM, DC-ELM, and CNN.

Method	*K*	LRF size Kernel size	Pooling size
DC-ELM	5	3 × 3	5 × 5
	10	3 × 3	5 × 5
	15	3 × 3	2 × 2

LRF-ELM	30	3 × 3	5 × 5

CNN	5	5 × 5	2 × 2
	10	5 × 5	2 × 2
	15	3 × 3	2 × 2

**Table 2:** Performance on MNIST (with only 10k training samples).

Algorithms	Training time (s)	Training accuracy		Testing accuracy	
		Mean	Std	Mean	Std
ELM	102.6	0.9965	0.0013	0.9457	0.0017
LRF-ELM	179.8	0.9882	0.0058	0.9763	0.0034
DC-ELM	**78.15**	0.9802	0.0027	**0.9803**	0.0015
CNN	2031.5	0.9703	0.0017	0.9724	0.0013
DBN	2968.4	0.9872	0.0022	0.9632	0.0021

**Table 3:** Performance on MNIST (with only 15k training samples).

Algorithms	Training time (s)	Training accuracy		Testing accuracy	
		Mean	Std	Mean	Std
ELM	169.3	0.9976	0.0012	0.9522	0.0009
LRF-ELM	264.9	0.9890	0.0039	0.9779	0.0021
DC-ELM	**120.4**	0.9887	0.0020	**0.9841**	0.0012
CNN	2960.9	0.9873	0.0014	0.9781	0.0011
DBN	4791.3	0.9905	0.0009	0.9759	0.0015

**Table 4:** Parameter setting for LRF-ELM, DC-ELM, and CNN.

Method	*K*	LRF size Kernel size	Pooling size
DC-ELM	10	3 × 3	5 × 5
	20	3 × 3	2 × 2

LRF-ELM	30	3 × 3	5 × 5

CNN	10	3 × 3	2 × 2
	20	2 × 2	2 × 2

**Table 5:** Performance on USPS (with 7000 training samples).

Algorithms	Training time (s)	Training accuracy		Testing accuracy	
		Mean	Std	Mean	Std
ELM	90.1	0.9974	0.0016	0.9323	0.0021
LRF-ELM	37.5	0.9849	0.0045	0.9651	0.0027
DC-ELM	**20.6**	0.9783	0.0034	**0.9693**	0.0028
CNN	2590.3	0.9750	0.0022	0.9614	0.0021
DBN	3861.4	0.9696	0.0033	0.9586	0.0026

**Table 6:** Performance on USPS (with 10000 training samples).

Algorithms	Training time (s)	Training accuracy		Testing accuracy	
		Mean	Std	Mean	Std
ELM	109.5	0.9992	0.0011	0.9650	0.0013
LRF-ELM	55.2	0.9948	0.0039	0.9803	0.0022
DC-ELM	**27.8**	0.9889	0.0025	**0.9843**	0.0014
CNN	3731.5	0.9815	0.0019	0.9736	0.0020
DBN	5284.1	0.9877	0.0024	0.9704	0.0015
